# Matched‐pair analysis of peri‐operative and oncological outcomes of robot‐assisted vs open retroperitoneal lymph node dissection

**DOI:** 10.1111/bju.16747

**Published:** 2025-04-22

**Authors:** Pailin Pongratanakul, Marieke Vermeulen‐Spohn, Carolin Wöltjen, Sophia Thy, Andreas Hiester, Peter Albers, Yue Che

**Affiliations:** ^1^ Department of Urology, University Hospital Düsseldorf Heinrich Heine University Düsseldorf Düsseldorf Germany

**Keywords:** germ cell tumours, retroperitoneal lymph node dissection, robotic surgery, oncological outcome, robot‐assisted RPLND, testicular cancer

## Abstract

**Objective:**

To analyse a comparatively large cohort of patients who underwent robot‐assisted retroperitoneal lymph node dissection (R‐RPLND) in a single centre, assessing the peri‐operative and oncological safety of this procedure compared to that in a matched‐pair cohort of patients who underwent open retroperitoneal lymph node dissection (O‐RPLND).

**Methods:**

We retrospectively identified 100 patients who underwent R‐RPLND between October 2010 and January 2024. A matched‐pair analysis of R‐RPLNDs and O‐RPLNDs was conducted based on the following criteria: surgical indication, histology, clinical stage (CS), and tumour size. The primary endpoint of this analysis was progression‐free survival (PFS). Secondary endpoints were peri‐operative parameters.

**Results:**

Based on surgical indication, the R‐RPLND cohort was divided into four groups: CS II seminoma (Group 1, 42 patients); marker‐negative CS II non‐seminoma (Group 2, 15 patients); CS I non‐seminoma with high‐risk factors (Group 3, seven patients), and post‐chemotherapy patients (Group 4, 34 patients). Two patients were excluded due to uncommon testicular histology. With a mean follow‐up of 32, 31, 32 and 28 months in the four groups, respectively, relapses occurred in 10/42 of Group 1, 3/15 of Group 2, and 1/7 of Group 3, while all patients remained relapse‐free in Group 4. The matched‐pair analysis revealed that histological retroperitoneal lymph node dissection specimens, relapse rates, and PFS were similar in the R‐RPLND and O‐RPLND groups. R‐RPLND had advantages in terms of a shorter hospital stay as a surrogate for less morbidity.

**Conclusion:**

In selected patients and selected surgical indications, R‐RPLND represents a minimally invasive alternative to O‐RPLND in the management of patients with testicular germ cell tumours.

**Abbreviations**
CS: clinical stage
GCT: germ cell tumour
NSGCT: non‐seminomatous germ cell tumour
O‐RPLND: open retroperitoneal lymph node dissection
PFS: progression‐free survival
RPLND: retroperitoneal lymph node dissection
R‐RPLND: robot‐assisted retroperitoneal lymph node dissection

## Introduction

Retroperitoneal lymph node dissection (RPLND) is a management strategy for patients with metastatic germ cell tumours (GCTs) [1, 2, 3]. After primary chemotherapy, RPLND is indicated for patients with non‐seminomatous GCTs (NSGCTs) with a marker‐negative residual tumour mass >1 cm [4, 5]. Patients with an initial metastatic NSGCT but negative markers may also be selected to undergo primary RPLND as an alternative to upfront systemic treatment, especially those with teratoma or adverse histological parameters in the primary tumour, such as somatic‐type mutations or sarcomatoid features [6, 7]. In addition, patients with initially clinical stage (CS) I GCTs but harbouring these adverse histological components in the primary tumour and high‐risk features, such as vascular invasion, may be selected for primary RPLND instead of adjuvant chemo‐ or radiotherapy. Further, several clinical trials have investigated the role and oncological outcome of primary RPLND in patients with CS II A/B seminomas [8, 9, 10, 11]. To date, open RPLND (O‐RPLND) represents the ‘gold standard’ of care; however, with advancements in robotic technology, there is an increasing interest in robot‐assisted RPLND (R‐RPLND) as a minimally invasive alternative. Multiple studies have already reported the feasibility and first oncological data on R‐RPLND [12, 13, 14, 15, 16, 17]. While many cohorts were small and/or multicentric, this study analysed a single‐centre cohort of 100 patients who underwent R‐RPLND. The aim of the analysis was to specify indications and selection criteria for R‐RPLND and to report oncological safety in a matched‐pair cohort of patients who underwent O‐RPLND to strengthen the existing evidence for R‐RPLND.

## Materials and Methods

### Study Population

To compare the long‐term oncological outcomes of patients who underwent R‐RPLND with those of a matched cohort of patients who underwent O‐RPLND, with the least possible bias. The O‐RPLND cohort was selected after matching with the R‐RPLND cohort based on surgical indication, clinical stage, primary histology, tumour size, and resection template.

We retrospectively reviewed our institutional testicular cancer database for all RPLNDs performed (*N* = 715) at the Department of Urology, University Hospital Düsseldorf. We identified 100 patients who underwent R‐RPLND and 59 matched patients who underwent O‐RPLND. Data on surgical performance, peri‐operative complications, and oncological outcomes were compared between the matched‐pair groups. While all of the R‐RPLNDs were performed in the period between October 2010 and January 2024, the most recent O‐RPLND in our matched‐pair cohort was performed on 9 August 2022. The analysis is presented in four separate subgroups based on surgical indication, as we believed this variable to have the greatest impact on outcome: CS II seminoma; marker‐negative CS II non‐seminoma; CS I non‐seminoma and high‐risk features (lymphovascular invasion or somatic‐type malignancy); and post‐chemotherapy RPLND. This study was approved by the local ethics committee (2019‐720) of University Hospital Düsseldorf of the Heinrich Heine University.

### Surgical Technique for R‐RPLND and O‐RPLND

All RPLNDs in this study were performed at the Department of Urology at University Hospital Düsseldorf. The robotic procedures were performed by four surgeons, with one senior mentoring the others to ensure consistency in technique. As a result, the surgical approach was standardised and directly comparable. The open procedures were also carried out by four different but highly experienced surgeons.

Our surgical technique for R‐RPLND has been previously described by our group [18]. The selection criteria for R‐RPLND at our institution are unilateral tumours no larger than 5 cm and no evidence of vascular invasion on imaging. All patients underwent unilateral modified template resections in a lateral flank position, with ipsilateral nerve sparing, if feasible. The ipsilateral ureter represented the caudal and lateral boundary of resection; the renal artery was described as the cranial boundary, and the crus of the diaphragm and psoas muscle as the posterior boundary. The lymphatic vessels were clipped and lymphatic tissue was removed using the ‘split and roll’ technique.

For the open approach, the procedure was performed via medial laparotomy with the patient positioned in a supine position. A retractor was used to optimise surgical exposure. The resection adhered to the same template boundaries as described above. Lymphatic vessels were clipped and lymphatic tissue was also removed using the split and roll technique.

### Endpoints

The primary endpoint was the evaluation of progression‐free survival (PFS) in the R‐RPLND group compared with the O‐RPLND group. PFS was defined as the time between the surgical procedure and date of relapse or date of last contact. The follow‐up protocol was based on the recommendations included in the European guidelines. A relapse was defined as either a radiological recurrence or biochemical evidence of disease progression. We assessed the percentage of relapses within each group and analysed the relapse pattern to identify potential implications for optimising future surgical techniques. Secondary endpoints included a comprehensive comparison of peri‐operative outcomes between the robotic and open approaches, focusing on operating time, blood loss, length of hospital stay, preserved ejaculatory function and intra‐operative as well as postoperative complications. Peri‐operative complications were categorised using the Satava and Clavien–Dindo classifications of intra‐operative and postoperative complications, respectively. Preserved ejaculatory function was assessed through patient history as part of tumour‐specific follow‐up care. It was documented as preserved if the patient reported the presence of antegrade ejaculation following the surgical procedure.

### Statistical Analysis

Statistical analyses were performed using SPSS (version 28.0; IBM Corporation, Armonk, NY, USA). Continuous variables are presented as median (interquartile range) or mean (sd). Categorical variables are presented as frequencies (*n*) and proportions (%). Data were compared between groups using Fisher's exact tests and *t*‐tests. A *P* value <0.05 was taken to indicate statistical significance. PFS was estimated using the Kaplan–Meier method.

## Results

A total of 100 patients were treated with R‐RPLND. Two patients were excluded from further analysis due to uncommon histology in the testis (Leydig or granulosa cell tumour). The remaining 98 patients undergoing R‐RPLND were categorised into four groups for further analysis, based on their surgical indication. Group 1 comprised 42 patients with CS II seminoma, Group 2 comprised 15 patients with marker‐negative CS II non‐seminoma, Group 3 comprised seven patients with CS I non‐seminoma and high‐risk features, and Group 4 comprised 34 patients with R‐RPLND after chemotherapy. From our database, we identified 59 patients undergoing O‐RPLND who were matched with the R‐RPLND group on surgical indication, primary histology, CS, tumour size, and resection template. Out of these 59 patients, 24 patients with CS II seminoma (Group 1B), six patients with marker‐negative CS II non‐seminomas (Group 2B), two patients with CS I non‐seminoma and high‐risk features (Group 3B), and 27 postchemotherapy cases (Group 4B) were identified. In Group 4, R‐RPLND was performed in 30 patients with CS II‐III non‐seminoma, two patients with pure seminoma with uncertain non‐seminoma content (due to elevated alpha‐fetoprotein), one patient with a seminomatous late relapse, and one patient with a pure seminoma with ureteric obstruction. The tumour was always unilateral, measuring <5 cm and marker‐negative in both approaches. Patients’ baseline characteristics are provided in Table 1.

**Table 1:** Clinical characteristics of patients undergoing robot‐assisted and open retroperitoneal lymph node dissection in the four groups.

Patient and tumour characteristics	Seminoma CS II		Non‐seminoma CS II		Non‐seminoma CS I, high risk		Post chemotherapy	
	Group 1 (*n* = 42)	Group 1B (*n* = 24)	Group 2 (*n* = 15)	Group 2B (*n* = 6)	Group 3 (*n* = 7)	Group 3B (*n* = 2)	Group 4 (*n* = 34)	Group 4B (*n* = 27)
Age, median (range) years	39 (35–49)	37 (28–53)	35 (31–47)	30 (21–39)	51 (33–59)		34 (27–42)	25 (18–49)
CS (preoperative), *n*								
I	0	0	0	0	7	2	0	0
II A	22	7	10	1	0	0	11	3
II B	20	17	5	5	0	0	14	10
II C	0	0	0	0	0	0	3	2
III	0	0	0	0	0	0	6	12
Prognostic group IGCCCG, *n*								
Good	42	24	15	6	7	2	28	19
Intermediate	0	0	0	0	0	0	5	6
Poor	0	0	0	0	0	0	1	2
Tumour markers (preoperative)								
AFP (µg/l)	2.3 (1.8–2.9)	2.9 (2.8–2.95)	3.2 (1.7–4.2)	2.15 (1.75–2.4)	4 (3.1–4.35)	4.4 (3.75–5.05)	3 (2.2–4.7)	2.4 (1.5–3.45)
β‐HCG (mU/ml)	0.4 (0.1–1.2)	0.1 (0.1–1.45)	2.4 (1.0–3.7)	0.1 (0.1–0.1)	0.55 (0.33–0.78)	0.1 (0.1–0.1)	0.2 (0.1–0.3)	0.1 (0.1–0.2)
LDH (U/l)	189 (171–239)	185 (162–198)	196 (181–225)	178 (173–187)	168 (161–172)	189 (170–208)	206 (180–229)	190 (164–203)
R‐RPLND template side, *n*								
Left	29	11	7	3	2	0	17	12
Right	13	13	8	3	5	2	17	15
Tumour size, cm								
Preoperative scan	2 (1.6–2.6)	2.25 (1.6–2.8)	1.7 (1.5–2.35)	2.3 (1.9–3.5)	0.5 (0.5–0.65)	1.25 (1.1–1.3)	1.5 (1.2–2.2)	3 (2.1–4)
RPLND histology	2.5 (1.9–3.8)	2.95 (2.7–4.1)	2.8 (1.85–3.2)	3.8 (2.75–4.2)	0.4 (0–0.65)	3.25 (3.225–3.275)	1.9 (1.5–3)	3 (2.25–4.6)
Testicular histology, *n*								
Pure seminoma	42	24	0	0	0	0	4	0
Non‐seminoma	0	0	15	6	2	0	30	27
Teratoma with somatic‐type malignancy	0	0	0	0	5	1	0	0
Other	0	0	0	0	0	1	0	0

Values are shown as median with interquartile range, unless otherwise stated.

AFP, alpha‐fetoprotein; CS, clinical stage; IGCCCG, International Germ Cell Cancer Cooperative Group; LDH, lactate dehydrogenase; RPLND, retroperitoneal lymph node dissection; R‐RPLND, robot‐assisted retroperitoneal lymph node dissection.

An oncological analysis was conducted based on the histological findings in the RPLND specimens and within the four groups (Table 2).

**Table 2:** Overview of histological retroperitoneal lymph node dissection specimens and oncological follow‐up data.

Oncological follow‐up	Seminoma CS II		Non‐seminoma CS II		Non‐seminoma CS I, high risk		Post chemotherapy	
	Group 1 (*n* = 42)	Group 1B (*n* = 24)	Group 2 (*n* = 15)	Group 2B (*n* = 6)	Group 3 (*n* = 7)	Group 3B (*n* = 2)	Group 4 (*n* = 34)	Group 4B (*n* = 27)
RPLND histology, *n* (%)								
Pure seminoma	38 (90)	22 (92)	7	0	0	0	0	0
Embryonal carcinoma	1 (2)	0	1	2	0	0	0	0
Mixed non‐seminoma	0	0	2	0	0	0	0	0
Teratoma	0	0	5	4	0	0	13 (38)	21 (78)
Necrosis/fibrosis/no tumour	3 (7)	2 (8)	0	0	7	2	21 (62)	6 (22)
Follow‐up, months								
Median (IQR)	25 (14.25–48.5)	46 (30–61)	24 (9.5–42.25)	56 (47.75–92)	32 (13.5–47)	59.5 (43.75–75.25)	21 (9–35)	95 (54.5–118)
Mean (sd)	32 (24.0)	48 (22.0)	31 (25.6)	83.75 (60.9)	32 (20.2)	59.5 (31.5)	28 (31.0)	93.5 (40.7)
Patients lost to follow‐up, *n*	0	1	1	2	0	0	9	11
Recurrences, *n* (%)	10 (24)	6 (26)	3	0	1	0	0	0
*P* value, two‐tailed	1.0		0.526		1.0		1.0	
Progression‐free survival								
Median (IQR)	20 (7.5–43.5)	31 (11.25–51)	17.5 (8.25–42.25)	56 (47.75–92)	32 (12.5–47)	59.5 (43.75–75.25)	21 (9–35)	95 (54.5–118)
Mean (sd)	27 (22.7)	32 (21.8)	28 (26.6)	83.75 (60.9)	32 (20.5)	59.5 (31.5)	28 (31.0)	93.5 (40.7)
Time to relapse								
Median (IQR)	10 (4.5–14.25)	6 (3.75–9)	5 (4.5–8)	x	10	x	x	x
Mean (sd)	13 (10.7)	7 (3.7)	7 (3.1)	x	10	x	x	x

CS, clinical stage; IQR, interquartile range; RPNLD, retroperitoneal lymph node dissection.

In Group 1, the RPLND specimens revealed seminoma in 38 patients (90%), embryonal carcinoma in one patient (2%), and no tumour in three patients (7%). In Group 1B, the RPLND specimens showed seminoma in 22 patients (92%) and no tumour in two patients (8%). The follow‐up duration was shorter in Group 1 (mean = 32 months) than in Group 1B (mean = 48 months). Recurrence rates were comparable in Group 1 (*n* = 10, 24%) and Group 1B (*n* = 6, 26%). The mean PFS was 27 months in Group 1 and 32 months in Group 1B. Relapses mainly occurred within the first two years, with a mean time to relapse of 13 months in Group 1 and 7 months in Group 1B. Analysing the recurrence pattern, Group 1 experienced six infield relapses (60.0%) and four outside‐field relapses (40.0%), whereas Group 1B experienced a higher proportion of outside‐field relapses (66.6%).

In Group 2, the RPLND specimens revealed vital tumours in 10 patients (67%), seminomas in seven patients (47%), embryonal carcinoma in one patient (7%), and mixed NSGCT in two patients (13%). Retroperitoneal histology revealed teratoma in the other five patients (33%). For Group 2B, the RPLND specimens revealed teratoma in four patients (67%) and embryonal carcinoma in two patients (33%). Regarding oncological outcomes, one patient in Group 2 and two patients in Group 2B were lost to follow‐up. The mean follow‐up duration was 31 months in Group 2 and 84 months in Group 2B, respectively. Relapses were observed only in Group 2 (*n* = 3, 21%) with a mean time to relapse of 7 months. The mean PFS was 28 months in Group 2 and 84 months in Group 2B.

In Groups 3 and 3B, the retroperitoneal specimens revealed no vital tumours because RPLND was performed preventively in patients with CS I with high‐risk features. The mean follow‐up time was 32 months in Group 3 and 59.5 months in Group 3B. One patient (14%) experienced a relapse 10 months after RPLND in Group 3, located outside‐field and involving mediastinal lymph nodes. The mean PFS was 32 months in Group 3 and 59.5 months in Group 3B.

In Groups 4 and 4B, the RPLND specimens revealed teratoma in 13 (38%) and 21 patients (78%), respectively. The remaining patients had necrosis. All patients remained progression‐free in both groups, with a mean follow‐up duration after RPLND of 28 months in Group 4 and 93.5 months in Group 4B.

With regard to surgical and peri‐operative outcomes (Table 3), the median operation times were similar for Group 1 (168 min) and Group 1B (150 min). Blood loss did not differ significantly between Group 1 (median = 0 mL) and Group 1B (median = 200 mL). Length of postoperative hospital stay differed significantly between Group 1 (median = 3 days) and Group 1B (median = 6 days; *P* < 0.01). Regarding functional outcome, antegrade ejaculation was preserved in 33 patients (97%) in Group 1 and 14 patients (93%) in Group 1B after excluding missing data. One patient reported retrograde ejaculation in each group. Intra‐operative complications occurred only in Group 1, with five patients (12%) experiencing vascular bleeding (Satava I), and one case (2%) of conversion to open surgery due to obesity. No intra‐operative complications were observed in Group 1B.

**Table 3:** Surgical outcomes and complications after robot‐assisted and open retroperitoneal lymph node dissection.

Peri‐operative variable	Seminoma CS II		Non‐seminoma CS II		Non‐seminoma CS I, high risk		Post chemotherapy	
	Group 1 (*n* = 42)	Group 1B (*n* = 24)	Group 2 (*n* = 15)	Group 2B (*n* = 6)	Group 3 (*n* = 7)	Group 3B (*n* = 2)	Group 4 (*n* = 34)	Group 4B (*n* = 27)
Operating time, min	168 (117–188)*	150 (150–165)*	150 (120–167)*	150 (150–180)*	150 (117–170)*	150 (150–150)*	198 (154–232)*	155 (150–180)*
*P* value, two‐tailed	0.74		0.98		0.69		0.37	
Estimated blood loss, mL	0 (0–50)*	200 (62.5–375)*	0 (0–50)*	50 (50–300)*	0 (0–100)*	250 (175–325)*	0 (0–50)*	155 (50–275)*
*P* value, two‐tailed	*P = 0.17*		*P = 0.24*		*P = 0.49*		*P = 0.068*	
Length of postoperative stay, days	3 (2–4)*	6 (4–6)*	2 (2–3)*	6 (4.5–6)*	2 (2–2)*	5 (4.5–5.5)*	3 (3–4)*	7 (6–8)*
*P* value, two‐tailed	< 0.01		< 0.01		0.23		< 0.01	
Antegrade ejaculation								
Yes	33 (97)^†^	14 (93)^†^	12 (100)^†^	3 (100)^†^	7 (100)^†^	2 (100)^†^	23 (88)^†^	16 (94)^†^
No	1 (3)^†^	1 (7)^†^	0	0	0	0	3 (12)^†^	1 (6)^†^
Missing	8	9	3	3	0	0	8	10
Intra‐operative complications								
Satava I	Vascular injury 5 (12)^†^	None	None	None	None	None	Vascular injury 3 (9)^†^	Vascular injury 3 (11)^†^
Satava II	Conversion 1 (2)^†^						Conversion 2 (6)^†^	
Postoperative complications								
Clavien–Dindo grade I		1 (4)^†^ 1× incisional fracture		None	None	None	3 (9)^†^ 1× chylous ascites 2× long recovery time	None
Clavien–Dindo grade II	1 (2)^†^ 1× transfusion							
Clavien–Dindo grade IIIa							rad.intervention 2 (6)^†^ 1× lymphocele 1× chylous ascites	
Clavien–Dindo grade IIIb	2 (5)^†^ 1× ureter lesion 1× lymphocele		1 (4.5)^†^ 1× lymphocele					
Clavien–Dindo grade IV		2 (8)^†^ 1× ileus 1× respiratory failure						

*P* values <0.01 indicate statistical significance.

CS, clinical stage.

* Values on the left are given in median (interquartile range).

^†^ Values on the right are *n* (%).

Postoperatively, one patient in Group 1 experienced a bleeding complication requiring transfusion after intra‐operative blood loss of 800 mL. There were significant postoperative complications requiring additional interventions in both groups. In Group 1, one case of lymphocele and one case of severe ureteric injury were reported, which could not be controlled by endoscopic approaches and ultimately required surgical revision with ureter replacement using an ileal interposition. In Group 1B, one minor postoperative complication (dehiscence of surgical wound) and two major complications were observed (ileus requiring re‐laparotomy with ileocecal resection and respiratory failure necessitating intensive care).

The median operation time was identical: 150 min in both Group 2 and Group 2B. Blood loss did not differ significantly between Group 2 (median = 0 mL) and Group 2B (median = 50 mL). Length of postoperative hospital stay differed significantly between Group 2 (median = 2 days) and Group 2B (median = 6 days; *P* < 0.01). Antegrade ejaculation was preserved in all patients in both groups. There were no intra‐operative complications in either group. Postoperatively, one case of lymphocele was observed in Group 2, and no complications were observed in Group 2B.

Comparison of RPLNDs for CS I non‐seminomas with high‐risk features showed that operating time was nearly identical, with a median time of 150 min in the two groups. No bleeding was reported in Group 3, but moderate blood loss (median = 250 mL) was observed in Group 3B, although the difference was not significant. The median length of postoperative hospital stay was 2 days in Group 3 and 5 days in Group 3B. All patients in both groups reported preserved antegrade ejaculation. No intra‐operative or postoperative complications were reported in either group.

In Groups 4 and 4B, the median operating time was 198 and 155 min, respectively. Blood loss did not differ significantly between Group 4 (median = 0 mL) and Group 4B (median = 155 mL). Length of postoperative hospital stay differed significantly between Group 4 (median = 3 days) and Group 4B (median = 7 days; *P* < 0.01). Preserved antegrade ejaculation was reported in 23 patients (88%) in Group 4 and 16 patients (94%) in Group 4B after excluding missing data. Intra‐operative complications occurred in both groups, with three cases (9%) of bleeding (Satava I) and two cases (6%) of conversion due to technical error of the robotic system and obesity (Satava II) in Group 4, while three cases (11%) of bleeding (Satava I) were observed in Group 4B. Postoperative complications were reported only for Group 4, with three minor complications (9%) including one patient with chylous ascites who was successfully managed by dietary restrictions and two patients with a longer recovery time. Two patients (6%) had Clavien‐Dindo grade IIIa complications, including lymphocele and chylous ascites requiring radiological intervention (e.g. drainage, lymphatic embolization).

## Discussion

With advances in robotic technology for urological malignancies, there is increasing interest in R‐RPLND, with multiple studies affirming its surgical feasibility [12, 13, 14, 15, 16, 17], consistent with our surgical outcomes. With increasing experience in robotic surgery and expertise in the surgical management of patients with testicular cancer, similar operating times and blood loss have been observed. R‐RPLND was associated with a significantly shorter hospital stay than O‐RPLND in our series, suggesting less postoperative pain and faster recovery owing to reduced morbidity from the surgical procedure. In both approaches, the overall complication rate was low. Nonetheless, in R‐RPLND, we observed a higher proportion of lymphoceles and chylous ascites, which might be attributable to less clipping in the learning curve for the robotic approach. These complications can be prevented by meticulously sealing the lymph vessels during the robotic dissection and can be well managed through dietary restrictions and, if necessary, radiological or surgical intervention.

**Fig. 1:**
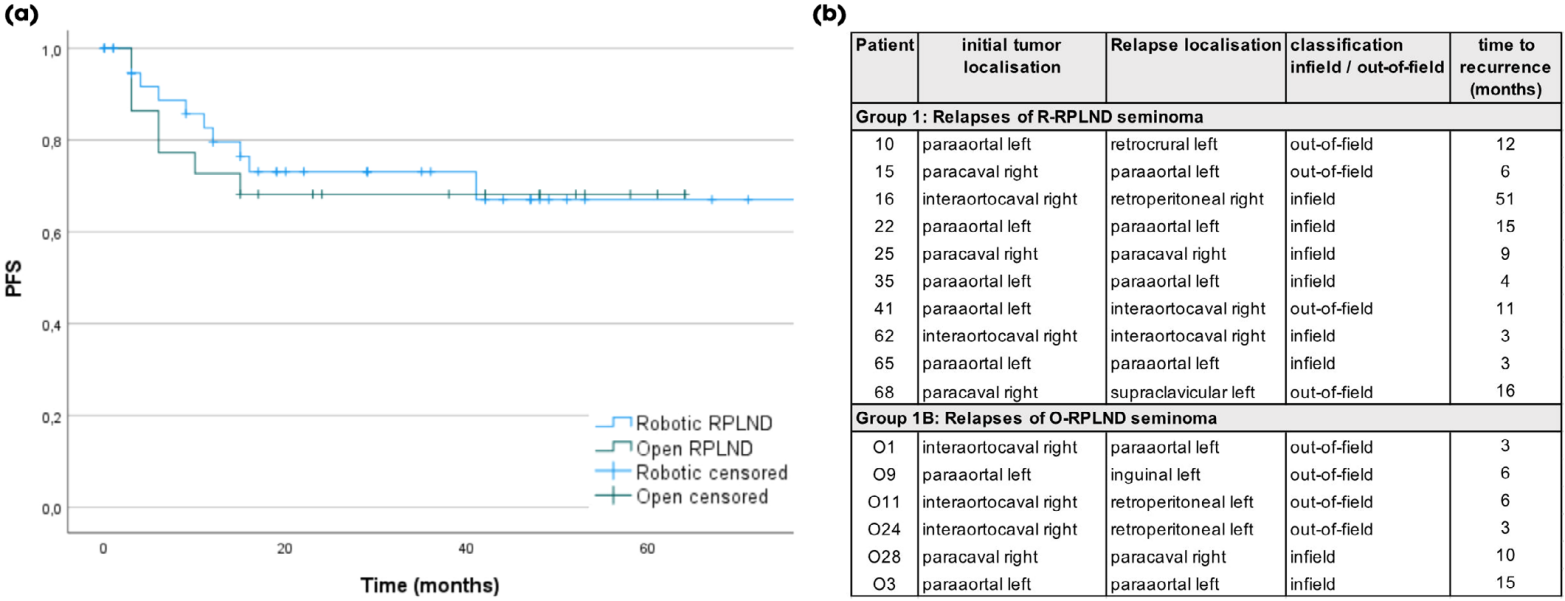
(**A**) Kaplan–Meier curves depicting progression‐free survival (PFS) of CS II seminoma patients after robotic and open retroperitoneal lymph node dissection. (**B**) Detailed description of seminoma recurrences after robotic and open retroperitoneal lymph node dissection.

The feasibility of R‐RPLND is well established, but oncological data remain limited. Recently, the first midterm oncological outcomes, with a follow‐up period of nearly 2 years, have been published for both primary and post‐chemotherapy R‐RPLND settings [16, 17]. Chavarriaga et al. [16] conducted a propensity‐matched analysis of primary R‐RPLND in a predominantly marker‐negative non‐seminoma cohort and reported a low relapse rate of 3.8% for the robotic and 7.8% for the open approach, with a median follow‐up of 23.5 months. In our study, we observed a higher recurrence rate in the primary RPLND setting, but our cohort consisted mainly of seminoma patients with predominantly CS IIB cases. We did not observe higher recurrence rates with R‐RPLND compared to O‐RPLND in patients with vital seminoma, suggesting that both techniques are equally effective in achieving oncological control (Fig. 1A). Although we report a relatively high proportion of infield recurrences in Groups 1 and 1B, we speculate that this may be related to the biological behaviour of the tumour rather than the surgical approach. For example, in the SEMS trial [11], although 35% of patients were treated with bilateral template O‐RPLND, 42% of total recurrences were in patients with bilateral template RPLND. Of these, 40% were infield recurrences. Nevertheless, the recurrence pattern after primary surgical resection of vital seminoma to avoid chemotherapy is a subject of further research at our institution (PRIMETEST II; ClinicalTrials.gov ID NCT06144736). Comparison of the pattern of recurrence between the robotic and open approaches showed that infield recurrences were more frequent with R‐RPLND compared to O‐RPLND (Fig. 1B), but not significantly so. In previous studies such as COTRIMS and PRIMETEST [8, 9], which included patients undergoing R‐RPLND and O‐RPLND, the recurrence pattern and number of infield recurrences did not differ.

In Group 2, 67% of the patients were found to have viable tumour cells in the retroperitoneal histology, mainly seminomas, despite the primary tumour being a non‐seminoma. While the other 33% had teratoma and, unsurprisingly, had a favourable oncological follow‐up, most patients with viable tumour cells also remained relapse‐free after surgery without adjuvant chemotherapy. Only three of the 15 patients experienced a relapse but were successfully treated with consecutive chemotherapy or subsequent surgery. Moreover, no recurrence was observed with O‐RPLND (Group 2B). These results underscore the effectiveness of surgical interventions and the potential to avoid adjuvant chemotherapy for a significant subset of patients.

Comparing the pattern of recurrence after R‐RPLND between different histologies, we observed more infield recurrences in patients with seminomas and no infield recurrences in those with non‐seminomas (Table 4). In the latter group, one patient experienced an outside‐field metastasis in the left parailiac site, while another showed an atypical recurrence spread, affecting the left iliac, retroperitoneal, and mesenteric regions. The first patient was successfully treated with further tumour resection and the second patient with chemotherapy. The third patient, who showed embryonal carcinoma in the RPLND specimen, exhibited a postoperative increase in beta‐hCG without radiological evidence of relapse and was managed with chemotherapy. These cases again highlight the different biological characteristics of seminomatous GCT metastases and may explain the observed discrepancy in the recurrence pattern after R‐RPLND. Seminomas show homogenous histology, whereas non‐seminomas often exhibit greater histological heterogeneity and may harbour more aggressive cell subpopulations with distinct tumour biology and behaviour [19], which may support dissemination to distant sites, leading to more outside‐field recurrences. In terms of recurrence pattern, atypical recurrence sites have been previously reported in five patients with testicular cancer after R‐RPLND [20], leading to the assumption of a peritoneal tumour seeding risk due to high intra‐abdominal pressure during the robotic approach. In our study we observed one patient with atypical intraperitoneal metastatic spread after primary R‐RPLND. The overall incidence of peritoneal seeding is low, since a multicentre study with 457 patients reported only two peritoneal‐type seeding events [14].

**Table 4:** Detailed description of recurrences in patients with non‐seminomas after robot‐assisted retroperitoneal lymph node dissection (Groups 2 and 3).

Patient	Primary testicular histology	R‐RPLND tumour histology and localisation	Relapse localisation	Classification infield/out‐of‐field	Time to recurrence, months	Further treatment
Group 2						
43	93% SEM, 5% EC, 2% CC	SEM: paraaortal left	Retroperitoneal, iliacal left, mesenterial	Atypical	5	3x BEP
50	100% EC	EC: paracaval right	β‐HCG increase postoperatively from 6 to 28 mU/ml	Biochemical	4	3x BEP
78	90% SEM, 10% TER	SEM: paraaortal left	Parailiacal/obturator left	Out‐of‐field	11	resection
Group 3						
84	40% EC, 20% YST, 20% CC, 20% TER	Interaortocaval right	Mediastinal right	Out‐of‐field	10	4x VIP

BEP, cisplatin, etoposide, and bleomycin; CC, chorioncarcinoma; EC, embryonal carcinoma; SEM, seminoma; TER, teratoma; VIP, cisplatin, etoposide, and ifosfamide; YST, yolk‐sac tumour.

Groups 3 and 3B underwent a preventive procedure, and no tumour was found in the RPLND specimens. In this subgroup, five of seven patients underwent R‐RPLND because of the presence of a teratoma with somatic‐type malignancy in the testicular primary. Only two patients underwent RPLND for lymphovascular invasion of the primary tumour. The recurrence rate was low, with only one patient in Group 3 (14%) experiencing a mediastinal recurrence. R‐RPLND is a particularly good alternative to O‐RPLND in this setting due to its minimal invasiveness.

Finally, in the post‐chemotherapy setting (Groups 4 and 4B), histological analysis of the RPLND specimen revealed teratoma in almost 40% of patients and all patients remained relapse‐free, underscoring the effectiveness of the multimodal treatment concept combining chemotherapy with residual tumour resection. This highlights the successful application of R‐RPLND, achieving comparable oncological results in highly selected patients. Our findings align with those of Ghoreifi et al. [17], who conducted a retrospective multicentre study of 159 post‐chemotherapy R‐RPLND cases. They reported eight recurrences (5%) after a follow‐up of 22 months. While the larger number of patients in their study provides robust validation, both datasets highlight the low recurrence rates achievable with the robotic approach, further supporting its role as a minimally invasive alternative to the conventional open approach with reduced morbidity.

This study had some potential limitations, including its retrospective design, covering data since 2010 involving four different surgeons, with most R‐RPLNDs performed in the last 2 years. Additionally, all R‐RPLNDs were performed in the lateral position with a modified template resection. Therefore, the oncological data may not be transferable to other centres performing mainly a RPLND with a bilateral template in the supine position. We have adopted the unilateral modified template technique for patients with low‐volume metastases at our centre, as recent publications have shown that the use of this technique achieves comparable results to bilateral template RPLND in terms of early oncological safety, and more favourable functional outcomes [18, 21, 22, 23, 24, 25].

Despite including 98 patients in total, the subgroups with robotic surgery were still small and more patients are needed to perform a comprehensive, robust oncological analysis. In particular Groups 2B and 3B included a very small number. We believe the reason for these small patient numbers is that, before R‐RPLNDs became commonplace in our department, patients with Group 2 features were mostly managed with image‐guided biopsies and patients with Group 3 features were mostly managed with active surveillance or adjuvant chemotherapy. This means that minimally invasive surgery has made it easier to consider surgery in cases where other alternatives are available.

In conclusion, in terms of oncological safety, R‐RPLND has been shown to be equivalent to O‐RPLND, offering less morbidity and a shorter hospital stay. However, more prospective, and ideally comparative, studies with longer follow‐up are needed to define this minimally invasive technique as a standard treatment for highly selected patients.

## Author Contributions

Each author participated sufficiently in the work to take public responsibility for all of the content and gave final approval of the submitted manuscript. The authors listed here made substantial contributions in the following areas: acquisition of data: Yue Che, Marieke Vermeulen, Pailin Pongratanakul, Sophia Thy, Andreas Hiester, Carolin Wöltjen; concept and design: Yue Che, Pailin Pongratanakul; statistical analysis: Pailin Pongratanakul; analysis and interpretation of data: Yue Che, Pailin Pongratanakul, Peter Albers; drafting of the manuscript: Pailin Pongratanakul, Yue Che; supervision: Yue Che, Peter Albers.

## Disclosure of Interests

All authors declare no conflicts of interest relating to this study.
